# Case of Poisoning by the Binoxalate of Potash

**Published:** 1842-11-19

**Authors:** 


*Case of Poisoning by the Binoxalate of Potash.*—A lady recently confined was ordered one ounce of the bitartrate of potash; but the apothecary sent by mistake that quantity of the binoxalate of potash, divided into two doses. One dose, or nearly half an ounce, was immediately swallowed, but had scarcely reached the stomach, when she was seized with excruciating pains, followed by violent convulsions, and death within ten minutes after swallowing the draught.

On opening the body twenty-four hours after death putrefaction was found to have commenced on all the lower parts of the abdomen. The mouth, the pharynx, and the œsophagus were in their natural state. The orifices of the stomach were hermetically closed, in consequence of the contraction of the circular fibres which surround them. The mucous membrane of the stomach presented the appearance of violent inflammation. The duodenum and small intestines presented the same appearances. The blood was fluid in the heart and great blood-vessels.

Another case is related where death also resulted from taking for three consecutive mornings a teaspoonful (holding about 1 drachm 16 grains) of the same salt. It produced severe vomiting after each dose, and death within an hour after taking the third dose. The body was not examined.—*Ibid, from Comptes Rendus des Seances de l'Acadamie des Sciences.* April, 1842.

